# Nystatin enhances the immune response against *Candida albicans* and protects the ultrastructure of the vaginal epithelium in a rat model of vulvovaginal candidiasis

**DOI:** 10.1186/s12866-018-1316-3

**Published:** 2018-10-25

**Authors:** Xu Zhang, Ting Li, Xi Chen, Suxia Wang, Zhaohui Liu

**Affiliations:** 1 Laboratory of Electron Microscopy, Ultrastructural Pathology Center, Peking University First Hospital, Beijing, 100034 China; 2 Department of Gynecology, Beijing Obstetrics and Gynecology Hospital, Capital Medical University, Beijing, 100026 China; 3 Department of Gynecology Minimally Invasive Center, Beijing Obstetrics and Gynecology Hospital, Capital Medical University, Beijing, 100026 China

**Keywords:** Vulvovaginal candidiasis, Vaginal epithelial cells, *Candida albicans*, Nystatin, Cytokines

## Abstract

**Background:**

Vulvovaginal candidiasis (VVC) is a common infectious disease of the lower genital tract. Nystatin, a polyene fungicidal antibiotic, is used as a topical antifungal agent for VVC treatment. The aim of the current study was to investigate the possible immunomodulatory effects of nystatin on the vaginal mucosal immune response during *Candida albicans* infection and examine its role in protection of vaginal epithelial cell (VEC) ultrastructure.

**Results:**

Following infection with *C. albicans*, IFN-γ and IL-17 levels in VECs were significantly elevated, while the presence of IgG was markedly decreased as compared to uninfected controls (*P* <  0.05). No significant differences in IL4 expression were observed. After treatment with nystatin, the level of IFN-γ, IL-17 and IgG was dramatically increased in comparison to the untreated group (*P* <  0.05). Transmission electron microscopy revealed that *C. albicans* invades the vaginal epithelium by both induced endocytosis and active penetration. Nystatin treatment protects the ultrastructure of the vaginal epithelium. Compared with the untreated *C. albicans*-infected group, Flameng scores which measure mitochondrial damage of VECs were markedly decreased (*P* <  0.001) and the number of adhesive and invasive *C. albicans* was significantly reduced (*P* <  0.01) after treatment with nystatin.

**Conclusions:**

Nystatin plays a protective role in the host defense against *C. albicans* by up-regulating the IFN-γ-related cellular response, the IL-17 signaling pathway and possibly through enhancing VEC-derived IgG-mediated immunity. Furthermore, nystatin notably improves the ultramorphology of the vaginal mucosa, partially through the protection of mitochondria ultrastructure in VECs and inhibition of adhesion and invasion by *C. albicans*. Together, these effects enhance the immune response of the vaginal mucosa against *C. albicans* and protect the ultrastructure of vaginal epithelium in VVC rats.

## Background

Vulvovaginal candidiasis (VVC) is a common infectious disease of the lower genital tract, which affects approximately 75% of women of reproductive age. Among patients with VVC, 6–9% suffer from recurrent VVC (RVVC) [1]. Most cases are caused by *Candida albicans*, one of the most common commensals, which acts as an opportunistic fungal pathogen in the vagina. The interactions between *C. albicans* and host defense mechanisms play an important role in determining whether colonization remains harmless or leads to epithelium infection. *C. albicans* adheres to, invades and damages epithelial cells. Host defenses of the vaginal epithelium include a mechanical barrier against invading pathogens and the local innate immune response triggered by *C. albicans* infection [2, 3].

Previous studies [4–6] have identified putative roles for several immune mediators in local host defenses against microbial infection. It is speculated that recognition of *Candida* can activate an epithelial cell-mediated cytokine response, leading to the recruitment and activation of various immune cells including neutrophils, dendritic cells and T cells [7, 8]. Interferon-gamma (IFN-γ) is a type II IFN produced by activated T cells, natural killer (NK) cells and natural killer T cells [9]. It activates phagocytes and favors the development of a Th1 protective response that participates in the clearance of fungal pathogens [10]. Expression of interleukin-4 (IL-4), the major cytokine involved in Th2 immune responses, correlates with disease exacerbation and pathology [11]. IL-17 has emerged as an essential mediator of protection against *C. albicans* in oral and dermal candidiasis [12]. It promotes antifungal immunity through up-regulation of pro-inflammatory cytokines, neutrophil-recruiting chemokines and antimicrobial peptides, which limit fungal overgrowth [13]. However, the role of IL-17-mediated immune responses in VVC is still controversial [14, 15]. In recent years, accumulating evidence has suggested that various non-B lineage cells including epithelial cells [16, 17] can produce immunoglobulin G (IgG). And the lymphoid-specific proteins RAG1 and RAG2, which are required for V(D)J recombination, are expressed in these cells. In addition, we previously showed that healthy vaginal epithelial cells (VECs) generated IgG in vitro [18]. However, the knowledge about the function of these non-lymphoid cell-derived Ig is still limited. Taken together, we speculate that Th2/Th1 balance may be related with antifungal activity against *C. albicans*.

Nystatin is an effective and broad-spectrum polyene fungicidal antibiotic which has been used for decades as a clinical antifungal agent for treating superficial candidiasis such as VVC [19, 20]. It functions mainly through binding to ergosterol and forming barrel-like, membrane-spanning channels in the plasma membrane of antibiotic-sensitive organisms [21, 22]. Increased permeability of fungi leads to leakage of small molecules and disturbance of cellular electrochemical components and subsequently fungal death [23]. In addition, nystatin can sequester cholesterol located on the plasma membrane of eukaryotic cells and alter the micro-structure of lipid rafts [24], which has been shown to play crucial roles in regulating both innate and adaptive immune responses through pathogen recognition, lymphocyte activation and cytokine signaling [25]. Therefore, nystatin may influence host antimicrobial defense responses. Several studies have focused on the putative immunomodulatory effects of nystatin. It is reported that nystatin up-regulates human macrophages chemokine CCL2 and CXCL8 levels [26]. Additionally, nystatin has been shown to act as a Toll-like cell receptor agonist [27], which induces immune responses by recruiting immune cells and promoting the secretion of chemokines [28, 29]. Whether nystatin can regulate the expression of immune modulators to promote an anti-*Candida* vaginal immune response has not yet been fully investigated.

Transmission electron microscopy (TEM) is a standard tool to investigate interactions between pathogenic fungi and the host due to its high resolution. To the best of our knowledge, nystatin-associated ultrastructural changes to the vaginal epithelium during *C. albicans* infection have not been investigated. The aim of the present study was to evaluate the possible antifungal mechanisms and immunoregulatory role of nystatin in VVC and to assess the protective effects on vaginal mucosa from an ultrastructural perspective.

## Methods

### Animals

The present study was approved by the Animal Ethics Committee (AEC) of Peking University First Hospital (PUFH). Specific pathogen free, non-mated, female Sprague-Dawley (SD) rats weighing 180–200 g were purchased from the Animal Center of Peking University Health Science Center. SD rats were housed in cages with controlled temperature (21 ± 2 °C) and humidity (30 ± 5%), and a 12/12 h light/dark cycle. A standard laboratory diet and free access to tap water were supplied. After adaptation for 1 week, the animals were anesthetized with 30 mg/kg of phenobarbital sodium, ovariectomized and maintained in pseudo-estrus state by subcutaneous injection of estradiol hexa-hydrobenzoate, 0.5 mg/week/rat, administrated as fractions three times a week until the end of the experiments [30].

### Microbial strains

*C. albicans* strains (ATCC-11006, provided by the Department of Dermatology laboratory, PUFH) were cultured aerobically on Sabouraud Dextrose Agar (SDA, Becton Dickinson, MD, USA) at 37 °C for 48 h. One colony of fungal cells was collected and suspended in RPMI 1640 and adjusted to 5.0 × 10^8^ yeasts/mL.

### Drug preparation

Nystatin vaginal effervescent tablets (Sino-American Shanghai Squibb Pharmaceuticals, Ltd.) consisted of 1.0 × 10^5^ units (100 mg) of nystatin per tablet and other auxiliary components. One vaginal effervescent tablet was dissolved in 5 ml of normal saline to prepare a drug solution of 2.0 × 10^4^ units/mL (20 mg/mL) and stored at − 20 °C for further use.

### Establishment of a VVC model in rats

Thirty SD rats were randomly divided into control (*n* = 6) and experimental groups (*n* = 24). Rats in the experimental groups were injected vaginally with 0.1 mL of *C. albicans* suspension (5.0 × 10^8^ yeasts/mL) and the control rats were injected with the same volume of RPMI 1640. The opening to the vaginas was blocked with aseptic cotton balls to prevent the outflow of fluid. At day 4 following inoculation, Gram staining of vaginal swabs from all rats was performed and examined by light microscopy (LM). Vaginal tissues biopsied from some of the infected rats were fixed in 4% paraformaldehyde, paraffin-embedded, sectioned and stained by hematoxylin-eosin (HE) to confirm inflammation. Rats with VVC were identified as those showing symptoms of inflammation and erythema, and having thick white vaginal discharges. Meanwhile, the presence of yeast and hyphae was confirmed by microscopy. Vaginal wash taken from each rat was cultured on SDA containing 50 mg/mL of chloramphenicol at 28 °C for 48 h. The number of yeasts of each animal was counted and expressed as colony-forming units (CFU)/mL at intervals during the vaginal infection.

**Fig. 1:**
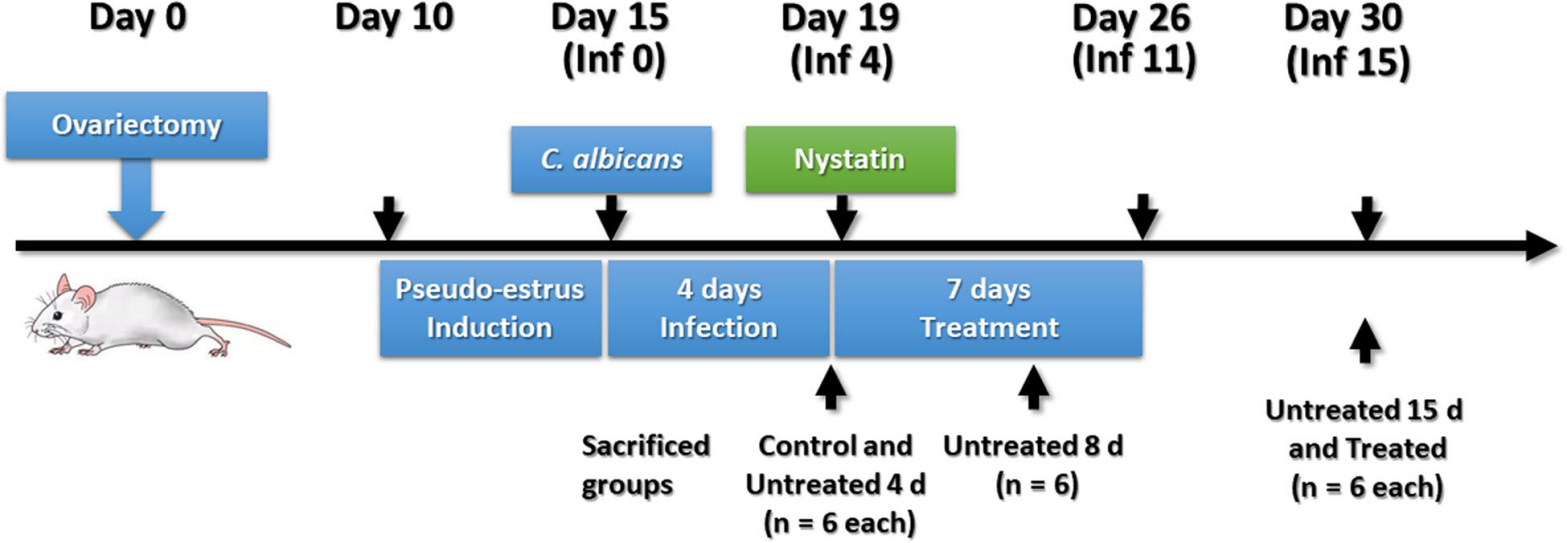
Schematic of experiments on *C. albicans* infection and nystatin treatment in rats. Inf represents Infection

After VVC confirmation, all rats in the experimental groups were randomly separated into nystatin-treated and untreated groups. To evaluate the time-dependent effects of *C. albicans* infection, rats in the untreated group were divided into 4 d, 8 d and 15 d sub-groups (*n* = 6 each group). Rats in treated group were injected vaginally with 2 × 10^4^ units/mL (20 mg/mL) of nystatin suspension for seven consecutive days. The dose of nystatin per day was determined according to animal equivalent dose calculations based on body surface area [31]. Meanwhile, the untreated rats received the same volume of normal saline vehicle (Fig. 1).

To determine drug efficacy of nystatin, the number of *C. albicans* (CFU) of vaginal wash was counted and the presence of hyphae or yeast of vaginal secretions was determined by Gram staining under LM in both nystatin-treated group and untreated 15 d sub-group. Samples with no detectable CFU together with no hyphae or yeast as determined by LM were defined as pathogen negative. The negative conversion rate (NCR) was calculated as the number of pathogen negative cases / 6 × 100%.

### Immunohistochemistry

**Fig. 2:**
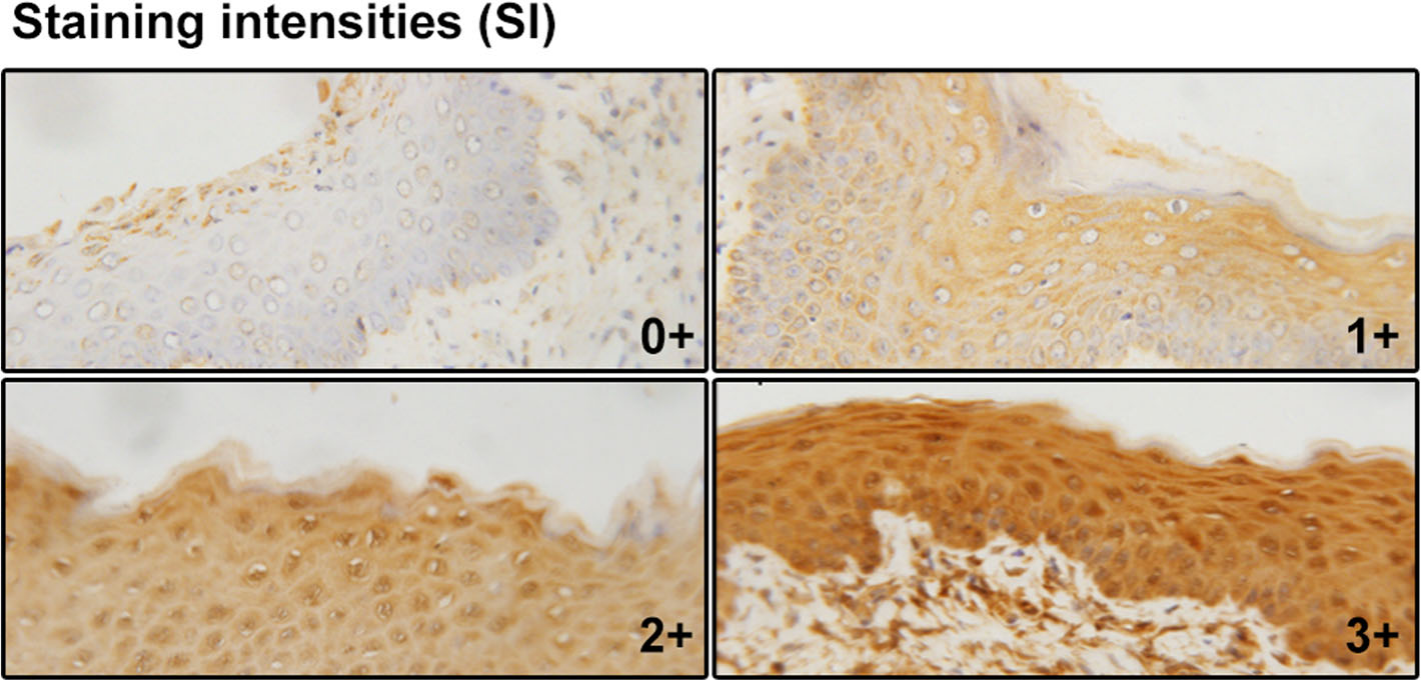
Overview of the staining intensities of immunoreactivity score (IRS). The staining intensities were shown (0+, 1+, 2+, 3+)

IFN-γ, IL-4, IL-17 and IgG levels were measured by immune labeling. Briefly, vaginal specimens were fixed in 4% paraformaldehyde, dehydrated in graded ethanol and embedded in paraffin. Deparaffinated sections 6 μm thick were incubated with 3% hydrogen peroxide to eliminate endogenous peroxidase, blocked with bovine serum albumin (BSA, TBD Science Technology, Tianjin, China) at room temperature for 10 min and then incubated overnight at 4 °C with anti-rat-IFN-γ, anti-rat-IL-4, anti-rat-IL-17 (rabbit polyclonal, Cloud-Clone, USA; 1:100 dilution for anti-rat-IFN-γ, 1:200 for anti-rat-IL-4 and 1:50 for anti-rat-IL-17) and anti-rat-IgG (RP125, provided by the Immunology Department, Peking University Health Science Center; 1:100 dilution), respectively. After rinsing with phosphate-buffered saline (PBS) three times, the sections were incubated with horseradish peroxidase conjugated anti-rabbit Ig (Zhongshan Golden Bridge Biotechnology). The negative control consisted of incubation with PBS instead of primary antibody. The results of immunohistochemistry were assessed by one experienced pathologist using a semi-quantitative analysis based on immunoreactivity score (IRS) [32]. The IRS-evaluation considers the percentage of stained area and visualized grade of color intensity by multiplying scores of staining percentage (SP; 0, lower than 10%; 1, 10–25%; 2, 26–50%; 3, 51–75%; 4, 76–100%) and staining intensity (SI; 0, negative; 1, mild; 2, moderate; 3, severe). The predominant grade of intensity was used as suggested previously [33] (Fig. 2).

### Transmission electron microscopy

Fresh rat vaginal tissues were fixed in 3% glutaraldehyde for 3 h and 1% osmic acid for 1 h at 4 °C. Following rinsing with PBS and dehydration in graded acetone, specimens were embedded in PON812 (SPI, West Chester, PA, USA) and polymerized at 60 °C for 24 h. Sections 70 nm thick were then double-stained with 5% uranyl acetate and lead citrate. Ultrastructural changes in different groups were observed by JEM 1230 TEM (JEOL, Japan). Quantification of adhesive and invasive *C. albicans*, in both yeast and hyphal forms, in nystatin treated and untreated 15 d sub-groups, was performed under a JEOL 1200 EX in 10 consecutive fields. Scoring of mitochondria in VECs according to the Flameng grading system [34] was performed using a JEOL 1200 EX. A total of five fields were randomly chosen from each slice, with each field including 20 mitochondria for semi-quantitative analysis. Each mitochondrion was scored as follows: 0, normal mitochondrial structure; 1, normal mitochondrial structure but missing particles; 2, mitochondrial swelling and transparent matrix without steep rupture; 3, steep rupture and concentrated matrix; 4, rupture, and incomplete inner and outer mitochondrial membranes.

### Statistical analysis

Statistical analysis was performed using SPSS version 19.0 (SPSS Inc., Chicago, IL, USA). Comparison of NCRs was carried out by Fisher’s exact test. All values are presented as the mean ± standard deviation unless otherwise indicated. Comparisons were performed using an independent sample *t* test, One-Way analysis of variance (ANOVA) and least significant difference (LSD) post hoc test. Calculated probabilities of 0.05 or less were considered to be statistically significant.

## Results

### Nystatin effectively treats VVC

**Fig. 3:**
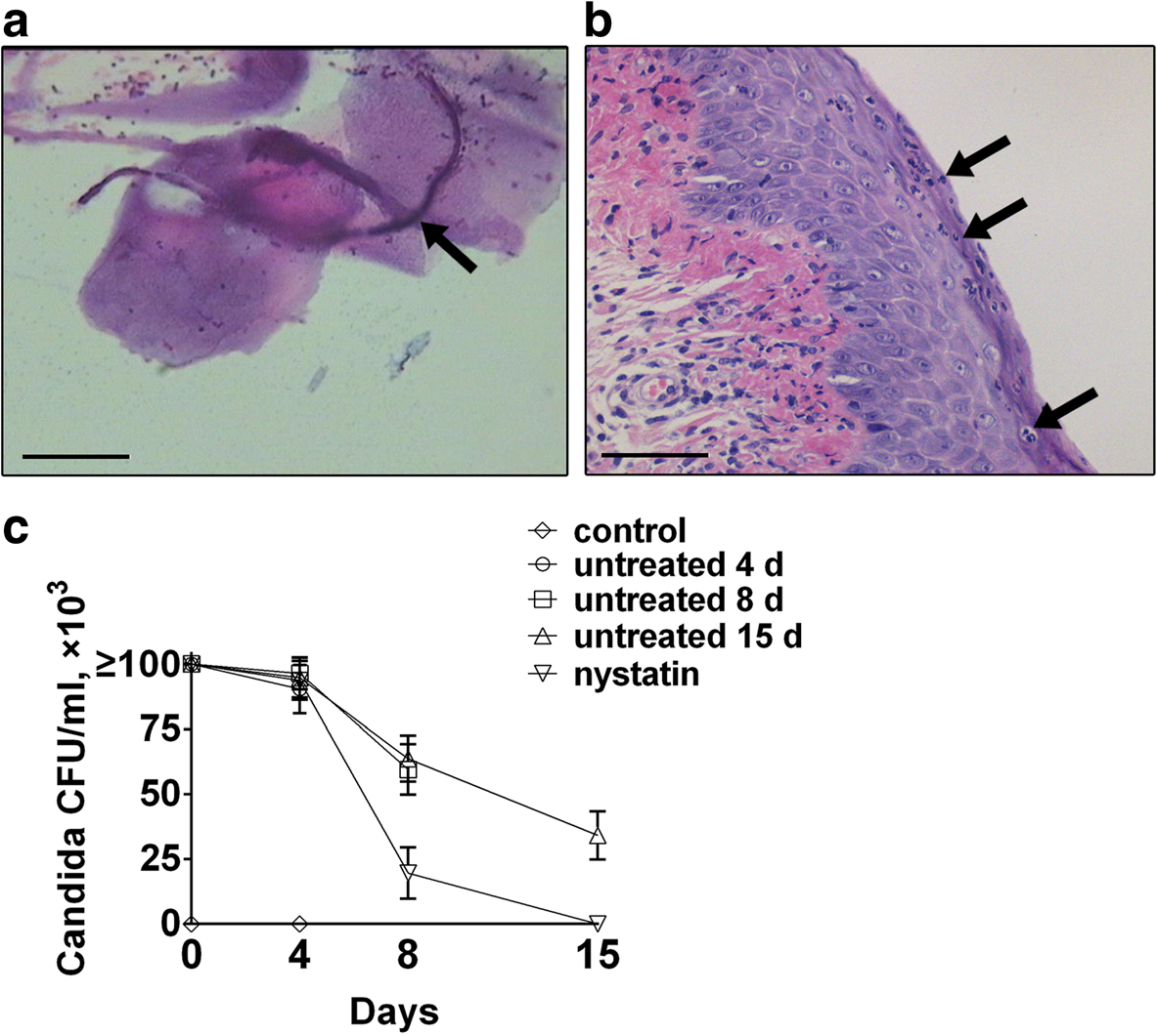
Confirmation of VVC model in rats and fungal burden from vaginal wash **a**, Light microscopy (LM) of Gram staining in a vaginal swab from rats infected by *C. albicans*. *C. albicans* hypha (arrow) adheres to vaginal epithelial cells. Scale bar = 20 μm, magnification × 1000. **b**, Representative image of inflammation of infected vaginal epithelium by Hematoxylin-eosin (HE) staining. Neutrophils (arrow) infiltrate the epithelium. Scale bar = 50 μm, magnification × 400. **c**, Outcome of vaginal fungal loads of rats infected with *C. albicans* (5.0 × 10^8^ yeasts/mL) in control, nystatin-treated and untreated 4 d, 8 d and 15 d sub-groups on days 0 (1 h post infection), 4 d, 8 d and 15 d post infection (*n* = 6 each group). The vaginal *C. albicans* colony-forming units (CFU) showed significant differences between in the nystatin-treated group and the untreated 15 d sub-group on both 8 d and 15 d post infection (All *P* < 0.001).Data represent the mean ± standard deviation

The status of VVC in infected groups and its control rats were evaluated on 4 d post infection. Neither symptoms of VVC nor hyphae and yeast in the vaginal swabs were observed in the control rats. Meanwhile, cultures of vaginal wash were also negative. In contrast, all infected rats exhibited inflammation, swelling and vaginal discharges. The presence of yeast and hyphae and inflammation was determined under LM (Fig. 3a, b). Following an intravaginal inoculation with *C. albicans*, > 1 × 10^5^ *Candida* CFU/mL were detected 1 h post infection and the number slightly declined to 9 × 10^4^ CFU/mL 4 d post infection (Fig. 3c). The successful rate of infection was 100%.

To evaluate the antifungal effects of nystatin, we observed symptoms, microscopic findings and vaginal fungal loads in rats. For the untreated 15 d sub-group, inflammation, swelling and vaginal discharges, as well as positive vaginal swabs and cultures of vaginal wash persisted in all cases. The NCR was zero. After administration of nystatin, all rats in the treated group achieved complete symptomatic relief. No hyphae or yeast were observed by LM. Furthermore, compared with the untreated 15 d sub-group, nystatin treatment caused a significant decline in yeast vaginal counts on infection day 8 (19.67 ± 9.873 vs 63.67 ± 8.824, *P* < 0.001) and 15 (0.00 ± 0.000 vs 34.17 ± 9.239, *P* < 0.001) (Fig. 3c). The NCR reached 100% (*P* = 0.001).

### Nystatin regulates the expression of cytokines in VECs

**Fig. 4:**
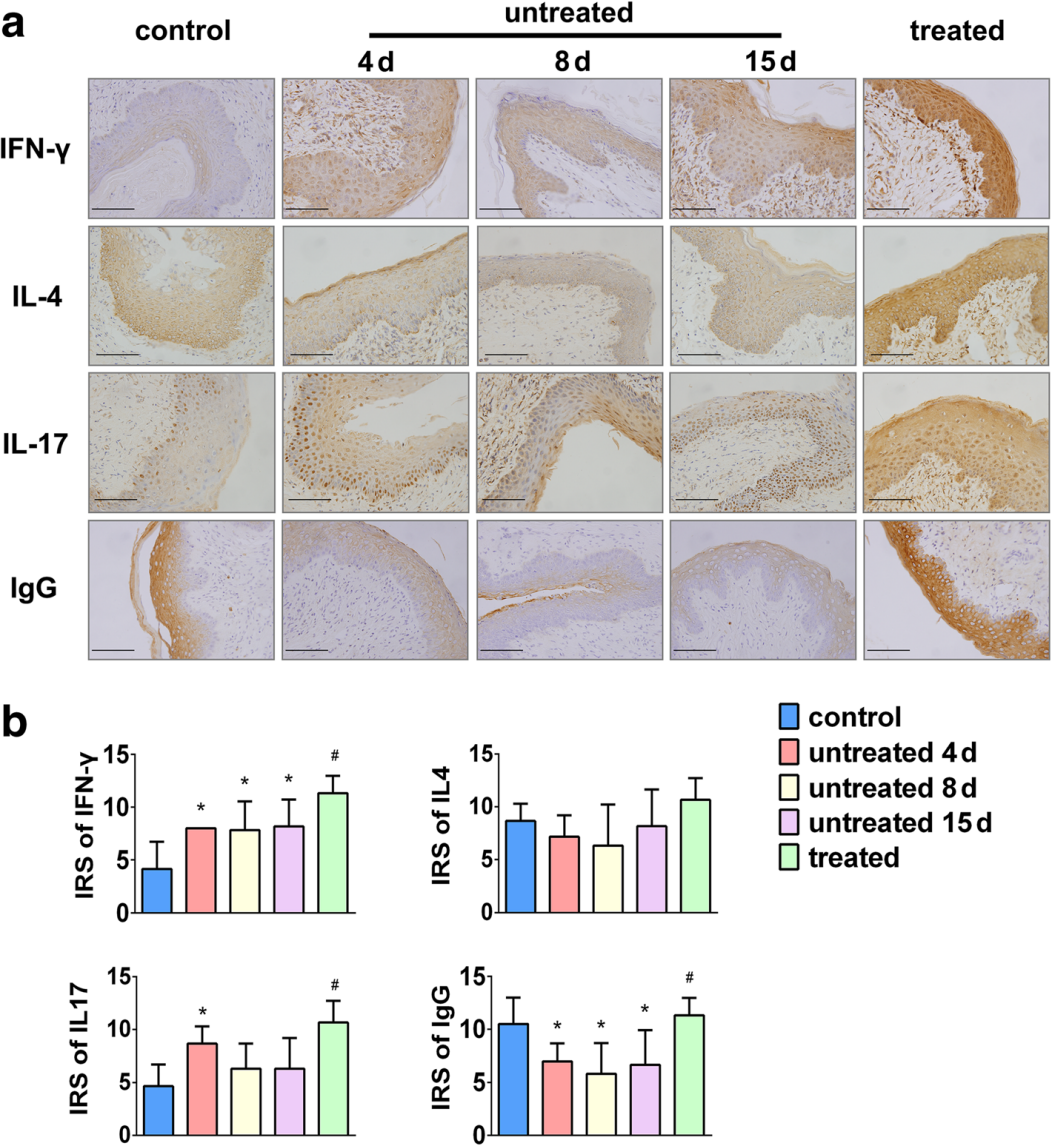
Effects of nystatin on the expression of cytokines and IgG in VECs in response to *C. albicans* infection*.* Rats in the untreated group were infected for either 4, 8 or 15 d with *C. albicans*. **a**, Representative images of immunolabeling of IFN-γ, IL-4, IL-17 and IgG in control, untreated and nystatin treated groups. Scale bar = 50 μm, magnification × 400. **b**, Semi-quantitative analysis revealed healthy VECs express low levels of IFN-γ, IL-4 and IL-17 and high level of IgG. A marked increase in the immunoreactivity score (IRS) of IFN-γ, IL-17 and a significant decrease of IgG were detected in untreated rats. After nystatin treatment, the IRS of IFN-γ, IL-17 and IgG was significantly up-regulated. No statistically significant differences were found in the levels of IL-4 expression among the different groups. Data represent the mean ± standard deviation (*n* = 6). * *P* < 0.05 compared with control group. ^#^ *P* < 0.05 compared with the untreated 15 d sub-group

To determine whether the expression of cytokines defined by the IRS differed in the uninfected controls, the infected nystatin-treated and untreated rats, immunohistochemistry was performed. Compared with the control group (4.17 ± 2.563), the expression levels of IFN-γ in the untreated group were significantly increased: 4 d sub-group (8.00 ± 0.000, *P* = 0.008), 8 d sub-group (7.83 ± 2.714, *P* = 0.011) and 15 d sub-group (8.17 ± 2.563, *P* = 0.006). The IFN-γ IRS in the treated group was significantly higher than that of the untreated 15 d sub-group (11.33 ± 1.633 vs. 8.17 ± 2.563, *P* = 0.029). No significant differences were observed among the untreated groups (Fig. 4).

As shown in Fig. 4, no statistically significant differences were found in the levels of IL-4 expression among the different groups. However, compared with the control group (8.67 ± 1.633), the IL-4 IRS of the untreated groups decreased: 4 d sub-group (7.17 ± 2.041, *P* = 0.384), 8 d sub-group (6.33 ± 3.882, *P* = 0.181) and 15 d sub-group (8.17 ± 3.488, *P* = 0.770). After nystatin treatment, the expression of IL-4 exhibited an increasing trend (treated group vs. untreated 15 d sub-group, 10.67 ± 2.066 vs. 8.17 ± 3.488, respectively, *P* = 0.162). We determined the Th2/Th1 balance by calculating the IRS ratios of IL-4/IFN-γ (Table 1). Compared with the control group, the Th2/Th1 IRS ratios in the untreated 4 d sub-group (*P* = 0.004), 8 d sub-group (*P* = 0.005) and 15 d sub-group (*P* = 0.009) were significantly decreased. No significant difference was detected between the untreated 15 d sub-group and nystatin-treated group (*P* = 0.840).

**Table 1:** IRS ratios of Th2/Th1 cytokines in control, untreated and nystatin-treated groups

Group	Th2/Th1 IRS ratio	*P*
Control	3.14 ± 2.57	
Untreated		
4 d	0.90 ± 0.26	0.004^a^
8 d	0.94 ± 0.70	0.005^a^
15 d	1.12 ± 0.62	0.009^a^
Treated	0.97 **±** 0.31	0.840^b^

The Th2/Th1 balance was determined by calculating the IRS ratios of IL-4/IFN-γ in different groups. ^a^*P* < 0.05 compared with control group. ^b^*P* > 0.05 compared with untreated 15 d sub-group. Data represent the mean ± standard deviation (*n* = 6)

The mean IRS for IL-17 in control rats was 4.67 ± 2.066. After infection with *C. albicans* for four days, the expression of IL-17 was significantly up-regulated (8.67 ± 1.633, *P* = 0.006). However, the IRS in the 8 d sub-group (6.33 ± 2.338, *P* = 0.219) and 15 d sub-group (6.33 ± 2.875, *P* = 0.219) showed no significant differences compared to control rats. Nystatin treatment stimulated a dramatic up-regulation in IL-17 compared with the untreated 15 d sub-group (10.67 ± 2.066 vs. 6.33 ± 2.875, respectively, *P* = 0.013). There were no statistically significant differences in IL-17 levels across the untreated groups (Fig. 4).

### Nystatin enhances IgG expression in vaginal epithelial cells

Compared with control rats (10.05 ± 2.510), the expression of IgG was significantly lower in the untreated groups: 4 d sub-group (7.00 ± 1.673, *P* = 0.033), 8 d sub-group (5.83 ± 2.858, *P* = 0.006) and 15 d sub-group (6.67 ± 3.266, *P* = 0.021). However, nystatin administration significantly up-regulated the levels of IgG expressed by VECs as compared to the untreated 15 d sub-group (11.33 ± 1.633 vs. 6.67 ± 3.266, respectively, *P* = 0.011). No significant differences were observed across the untreated groups (Fig. 4).

### Nystatin protects the infected vagina against *C. albicans*

To further investigate the interactions between *C. albicans* and VECs, as well as host defense mechanisms and the protective effects of nystatin, we performed TEM to observe the adhesion and invasion of the fungal pathogen into the vaginal epithelium, as well as ultrastructural restoration in treated rats.

**Fig. 5:**
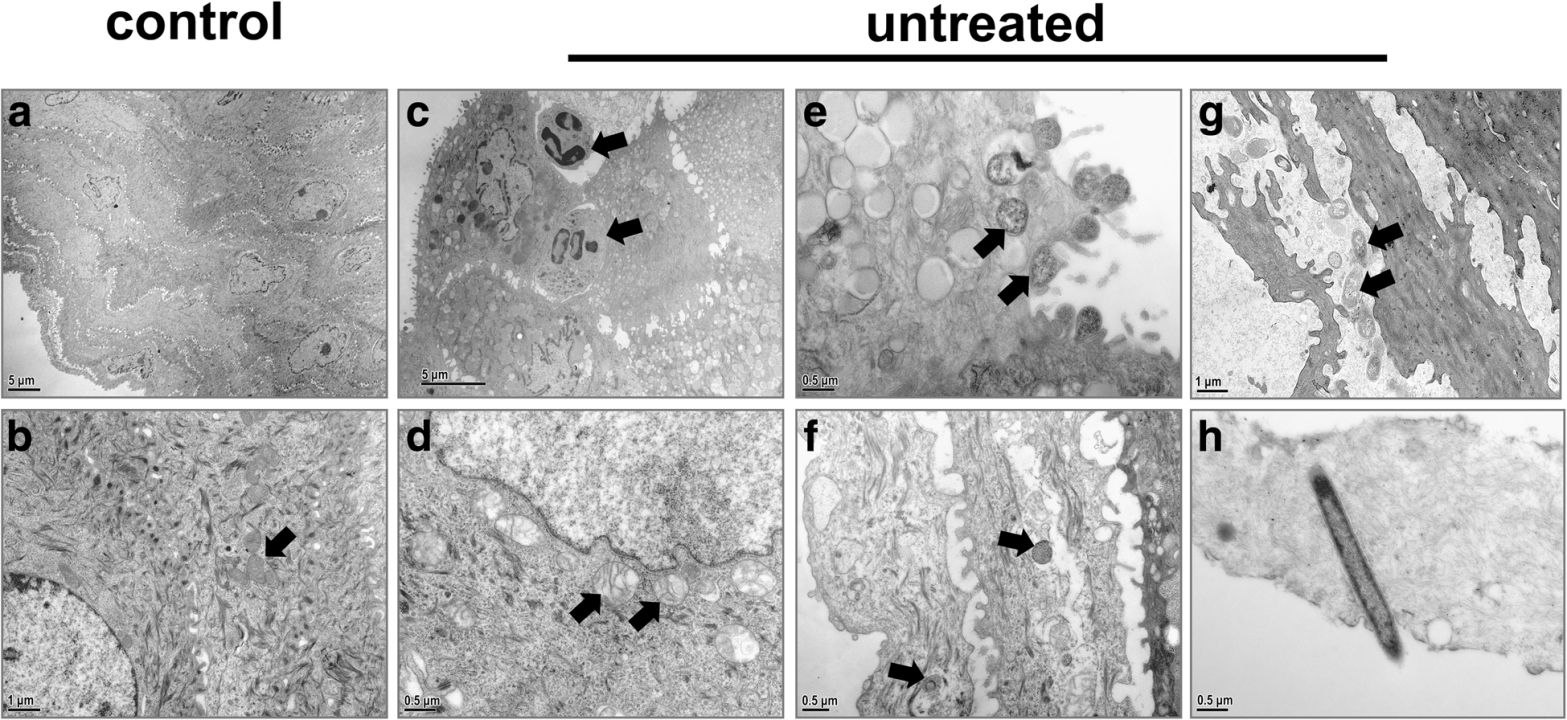
Ultrastructure of the vaginal epithelium in the control group and untreated 4 d and 8 d sub-groups in the early stages of *C. albicans* infection. **a**, Rats in the control group show normal vaginal morphology with a non-keratinized stratified squamous epithelium. Magnification × 3000. **b**, Adjacent VECs were bounded together by desmosomes and mitochondria (arrow) with normal morphology were observed in VECs from control rats. Magnification × 15,000. **c**, A large number of neutrophils (arrow) infiltrate into the vaginal epithelium after *C. albicans* infection. Magnification × 6000. **d**, Mitochondria (arrow) in VECs are mildly or moderately swollen with irregularly arranged cristae and decreased matrix density. Magnification × 30,000. **e**, VEC pseudopods enveloped yeasts. Arrows indicate yeasts integrated with VECs. Magnification × 30,000. **f**, Yeasts (arrow) invaded into the superficial layers of the vaginal mucosa. Magnification × 25,000. **g**, *C. albicans* hyphae (arrow) observed on the surface of the vaginal epithelium. Magnification × 15,000. **h**, A hypha penetrated into fiber-like keratinized materials of the VEC surface. Magnification × 30,000

**Fig. 6:**
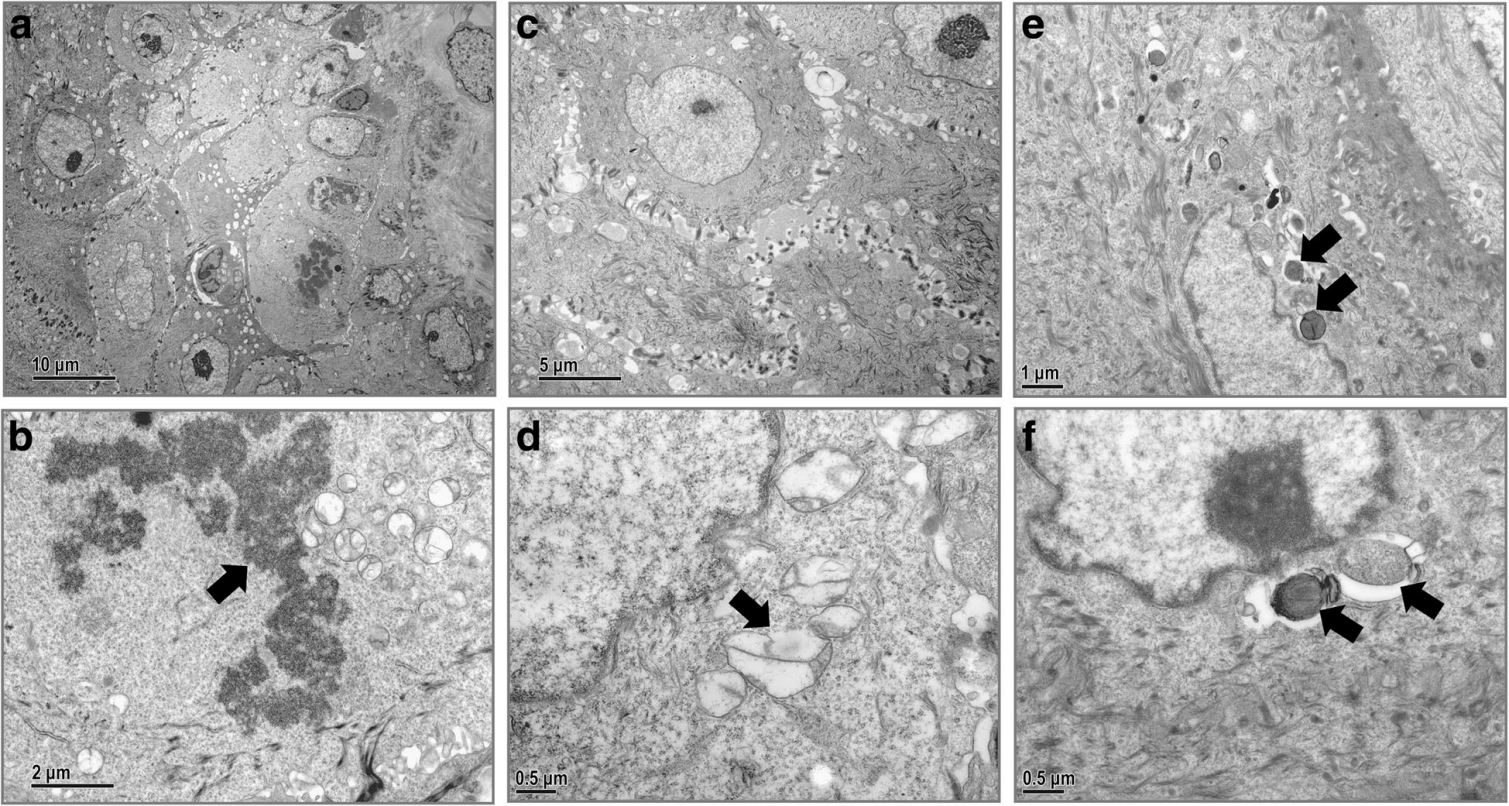
Ultrastructural changes in the vaginal epithelium infected with *C. albicans* in the untreated 15 d sub-group. **a**, The vaginal mucosa is extensively damaged and inflammatory cells have infiltrated the vaginal epithelium. Magnification × 3000. **b**, Karyolysis with dissolved chromatin (arrow) of the VECs. Magnification × 15,000. **c**, Desmosomes between adjacent epithelial cells destroyed and intercellular spaces widened. Magnification × 6000. **d**, Mitochondria in VECs significantly swollen with decreased matrix density and incomplete rupture of the inner and outer mitochondrial membranes (arrow). Magnification × 30,000. **e**, More yeasts (arrow) are observed in the epithelial cytoplasm. Magnification × 15,000. **f**, The yeasts (arrow) in VECs are of irregular shape and appear to reproduce by budding. Magnification × 30,000

**Fig. 7:**
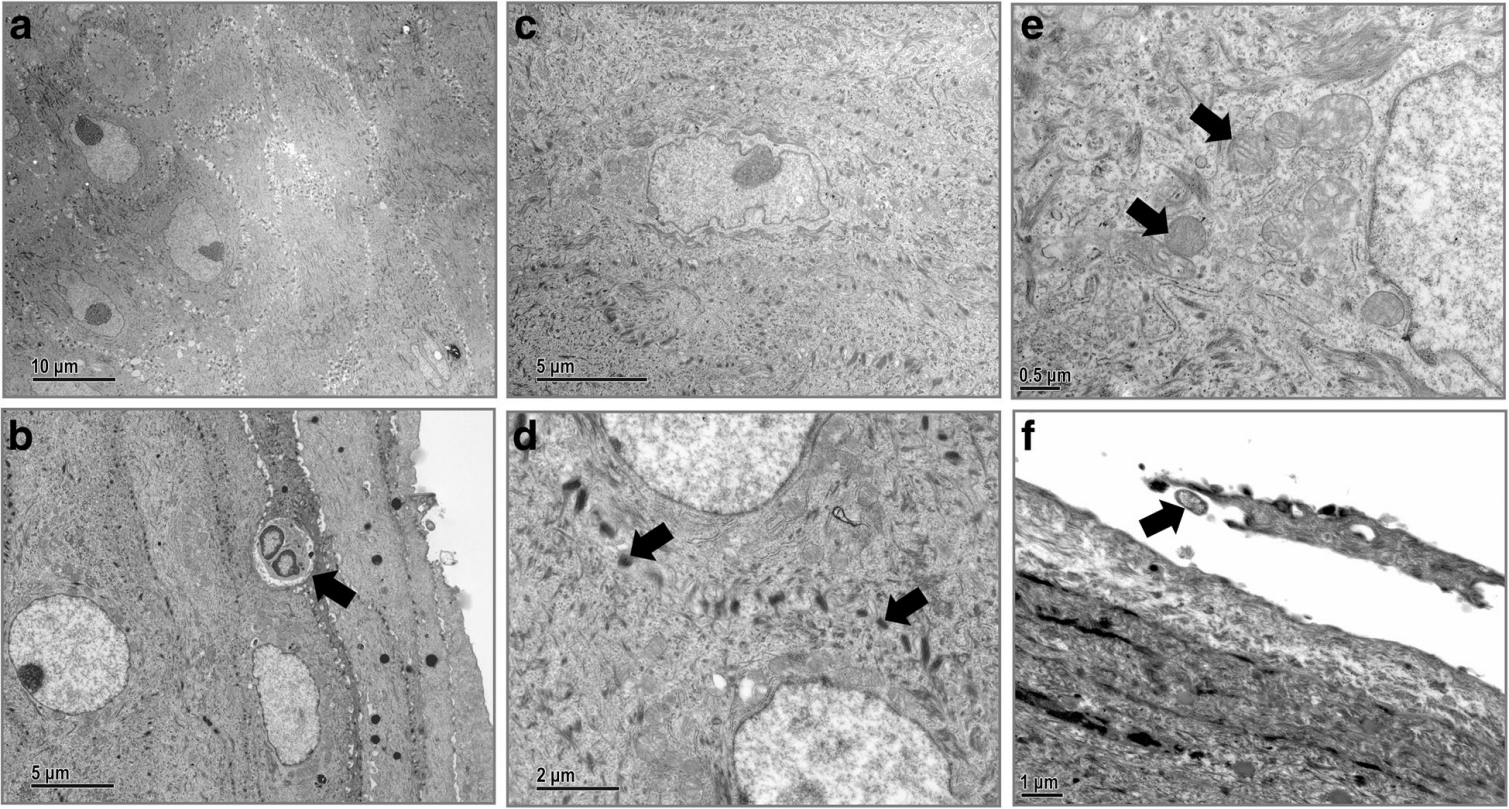
Nystatin protects the ultrastructure of VECs from *C. albicans* infection. **a**, The vaginal epithelium was restored after nystatin treatment. Magnification × 3000. **b**, Scattered neutrophils (arrow) observed in superficial VECs. Magnification × 6000. **c**, The intercellular space between VECs is restored. Magnification × 8000. **d**, The morphology of VEC junctions (arrow) is restored. Magnification × 15,000. **e**, The ultrastructure of mitochondria (arrow) in VECs is restored to normal or showed only mild swelling. Magnification × 30,000. **f**, Few hyphae (arrow) are detected on the surface of VECs. Magnification × 15,000

The vaginal mucosa in control rats consisted of non-keratinized stratified squamous epithelium with desmosomes that bound adjacent cells together (Fig. 5a, b). The epithelial cells showed abundant tonofibrils and glycogen, and mitochondria with normal structures were distributed in the cytoplasm (Fig. 5b). Following infection with *C. albicans*, the vaginal mucosa of untreated rats exhibited ultrastructural impairment. In the early stage of infection, numerous neutrophils were recruited and infiltrated the vaginal epithelium and lamina propria (Fig. 5c). Superficial epithelial cells displayed a large number of lipid droplets, mucus granules and decreased glycogen deposits. The mitochondria in VECs were mildly or moderately swollen with decreased matrix density (Fig. 5d). Elongated finger-like and/or bubble-like pseudopods protruded from the surface of VECs. The pseudopods contacted with *C. albicans* yeast cells and appeared to engulf these organisms (Fig. 5e). In addition, yeasts and hyphae penetrated superficial layers of the vaginal mucosa (Fig. 5f-5h). At infection day 15, the entire epithelial layer was destroyed (Fig. 6a). Epithelial cells exhibited a shrunken morphology characteristic of necrosis (Fig. 6b), resulting in damage to desmosomes and increased intercellular spaces (Fig. 6c). Mitochondria in VECs were markedly enlarged, with transparent matrix, ruptured cristae and incomplete inner and outer mitochondrial membranes (Fig. 6d). A greater number of yeasts were observed in the cytoplasm of VECs at day 15 compared to the early stage of infection (Fig. 6e). The yeasts are irregular shaped and seem to reproduce by budding (Fig. 6f). In the nystatin-treated group, the ultrastructure of the vaginal epithelium was notably improved (Fig. 7a). Few scattered inflammatory cells were observed in the entire layer of mucosa (Fig. 7b). The width of the intercellular spaces decreased and no damage was observed at cell junctions (Fig. 7c, d). The morphology of the mitochondria in VECs was normal or showed only slight swelling (Fig. 7e). Furthermore, compared with the untreated 15 d sub-group, nystatin treatment significantly reduced the number of adhesive and invasive *C. albicans* in both yeast and hyphal forms respectively (Table 2, Fig. 7f).

**Table 2:** Quantification of adhesive and invasive *C. albicans* in nystatin-treated and untreated groups

	Untreated	Treated	*t*	*P*
Adhesive yeasts	2.50 ± 1.049	0.00 ± 0.000	5.839	0.002
Adhesive hyphae	9.50 ± 1.049	1.50 ± 0.548	16.562	< 0.001
Invasive yeasts	12.33 ± 1.966	2.50 ± 1.049	10.808	< 0.001
Invasive hyphae	1.33 ± 0.516	0.00 ± 0.000	6.325	0.001

The number of adhesive and invasive *C. albicans* in the nystatin-treated group and untreated 15 d sub-group was evaluated by counting 10 consecutive fields under transmission electron microscopy (magnification ×1200). Data represent the mean ± standard deviation (*n* = 6)

The ultrastructure of the mitochondria is regarded as an early morphological indicator of cell damage. Therefore, we determined the Flameng scores of mitochondria in VECs among different groups. As shown in Table 3, the Flameng scores of mitochondria in untreated 4 d, 8 d and 15 d sub-groups were significantly higher as compared with the control group (all *P* < 0.001). Furthermore, in untreated sub-groups, a significant increase in Flameng score was observed with prolonged *C. albicans* infection (all *P* < 0.001). Administrated of nystatin significantly decreased the Flameng scores of mitochondria in VECs (*P* < 0.001).

**Table 3:** Flameng scores of mitochondria in VECs in control, untreated and nystatin treated groups

Group	Flameng score	*P*
Control	0.13 ± 0.342	
Untreated		
4 d	0.72 ± 0.636	*P* < 0.001^a^
8 d	1.94 ± 0.904	*P* < 0.001^a, b^
15 d	3.10 ± 0.542	*P* < 0.001^a, b, c^
Treated	0.51 ± 0.707	*P* < 0.001^d^

Flameng scores of mitochondria in VECs were assessed by transmission electron microscopy for different groups. ^a^*P* < 0.001 compared with control group. ^b^*P* < 0.001 compared with untreated 4 d sub-group. ^c^*P* < 0.001 compared with untreated 8 d sub-group. ^d^*P* < 0.001 compared with untreated 15 d sub-group. Data represent the mean ± standard deviation (*n* = 6)

## Discussion

The vaginal mucosa serves as the first line of defense against *C. albicans* through its anatomical and physiological characteristics, including the physical barrier of the epithelium, the shedding and renewal of VECs, and the vaginal pH. It has been shown that recognition of *C. albicans* by epithelial cells induces a series of cytokines, which is immensely important in innate immune defense and pathogen elimination [7, 35]. Our present study shows that healthy VECs express low levels of IFN-γ, IL-4 and IL-17. After *C. albicans* infection, expression of IFN-γ and IL-17 increases. In addition, large number of neutrophils are observed in the epithelium, suggesting that activation and recruitment of immune cells into the mucosal layer might be involved in the pathogenesis of VVC. Elevated levels of IFN-γ and IL-17 indicate that host immune defenses are activated in the early stages of *C. albicans* infection. Furthermore, we noted that IFN-γ and IL-17 levels are remarkably increased with nystatin treatment. We speculate that a relatively high level of cytokines is a prerequisite for activating a protective mucosal response or maintaining homeostasis [36], whereas cytokine levels in untreated epithelium failed to further enhance the initiated immune processes. Therefore, we propose that nystatin could play an immunoregulatory role by increasing IFN-γ and IL-17 levels to enhance vaginal anti-fungal immunity.

Previous studies [37, 38] have demonstrated that the balance of Th2/Th1 cytokines is involved in the pathogenesis of VVC and RVVC, determining susceptibility to *C. albicans* and the symbiotic relationship between the mucosa and pathogens. Generally, Th2 cytokines participate in the humoral immune response associated with enhanced vulnerability to pathogenic infection, while the Th1 cytokine response induces protective mucosal immunity. IL-17 is also essential for mucosal defense against *C. albicans* by recruitment of neutrophils and induction of antimicrobial peptides [39, 40]. Based on our results, in early stages of *C. albicans* infection, there is a significant decrease in the Th2/Th1 ratio and an increase in IL-17 in VECs. This suggests that the VEC-mediated local cellular immune response is enhanced and a rapid secretion of IL-17 by VECs is involved in protection against *C. albicans*. With infection time prolonged, it was noted that the ratio of Th2/Th1 on infection day 15 increased with tissue deterioration as observed ultrastructurally, suggesting a decreased clearance of mucosal *C. albicans* with suppressed immunity. However, whether chronic infection after 15 d would bring the ratio back to uninfected levels still need to be confirmed. In contrast to the untreated group, nystatin treatment again shifts balance to Th1 with a comparable Th2/Th1 ratio and elevated IL-17 level. These findings suggest that nystatin might provide a protective stimulation by up-regulating the expression of Th1 cytokines and promote a Th17-biased mucosal immune response to eliminate vaginal *C. albicans*.

IgG plays a vital role in the humoral immune response through antigen recognition, complement binding and activation of effector cells. It has traditionally been believed that the production of Ig molecules is restricted to B lineage cells. However, Ig genes and proteins have been recently found in a variety of types of cells, including proliferating epithelial cells [16, 17]. The Ig molecules expressed by these cells consist predominantly of IgG, and the light chains expressed are mainly kappa chains. Additionally, the functions of epithelial cell-origin Ig molecules are still unknown. In the present study, normal rat vaginal epithelium expressed a high level of IgG. The presence of IgG decreased following infection with *C. albicans* but was significantly up-regulated following nystatin treatment. Although, it is likely that VEC-derived IgG may be involved in local immune response, the antifungal role of non-lymphoid-origin IgGs is not unequivocally established yet. Further investigations are needed to confirm whether VEC-derived IgG participates in the antifungal immunity and the protective mechanisms of nystatin.

Mitochondria are involved in cellular events such as metabolism, bioenergetics, cell death, and innate immune signaling [41]. Fungal infections are associated with alterations in cellular physiology, which directly or indirectly impairs mitochondrial ultramorphology. An accumulation of injured mitochondria activates a vicious cycle of mitochondrial damage and cell death [42]. Generally, *C. albicans*-induced VEC damage is characterized by mitochondrial swelling [36]. In the current study, our ultrastructural findings provide evidence that *C. albicans* invades VECs through induced endocytosis and active penetration, which is consistent with our previous in vitro observations by scanning electron microscopy [18]. In our study, a prolonged infection time resulted in deterioration of the ultrastructure of the vaginal mucosa in untreated rats and altered morphology of mitochondria in VECs. However, with administration of nystatin, we observed a significant decrease in both intra- and extracellular *C. albicans*. Furthermore, the ultrastructure of the vaginal epithelium and mitochondria in VECs was significantly improved. These ultrastructural observations indicate that the protective anti-fungal effects of nystatin on the vaginal mucosa in VVC rats are partially associated with its capacity to protect mitochondrial morphology and inhibit adhesion and invasion by the fungal pathogen.

## Conclusions

Our current study indicates that nystatin plays a protective role in the host defense against *C. albicans* by: (1) activating functional cytokines through up-regulation of IFN-γ and IL-17 signaling; (2) significantly increasing the expression of VEC-derived IgG and likely modulating the antibody-mediated pathway; (3) exerting fungicidal effects by inhibiting adhesion and invasion of *C. albicans*; and (4) shielding the ultrastructure of the vaginal epithelium partially through protecting the morphology of mitochondria in VECs. Further studies are needed to investigate the molecular mechanisms of nystatin-mediated regulation of the vaginal epithelial immune response during VVC in more detail.

## Abbreviations

AEC: Animal Ethics Committee
ANOVA: One-Way analysis of variance
BSA: Bovine serum albumin
*C. albicans*: *Candida albicans*
CFU: Colony-forming units
HE: Hematoxylin-eosin
IFN-γ: Interferon-gamma
IgG: Immunoglobulin G
IL-17: Interleukin-17
IL-4: Interleukin-4
IRS: Immunoreactivity score
LM: Light microscopy
LSD: Least significant difference
NCR: Negative conversion rate
NK: Natural killer
PBS: Phosphate-buffered saline
PUFH: Peking University First Hospital
RVVC: Recurrent vulvovaginal candidiasis
SD: Sprague-Dawley
SDA: Sabouraud Dextrose Agar
SI: Staining intensity
SP: Staining percentage
TEM: Transmission electron microscopy
VECs: Vaginal epithelial cells
VVC: Vulvovaginal candidiasis
